# The four killers of Meckel's diverticulum

**DOI:** 10.4103/0974-2700.50754

**Published:** 2009

**Authors:** El Bachir Benjelloun, Amal Ankouz, Khalid Mazaz, Khalid Ait Taleb

**Affiliations:** Department of General Surgery, UH Hassan II of Fez, Morocco

Sir,

A 28-year-old woman presented to the Emergency Department (ED) with complaints of abdominal pain, vomiting and constipation since the last 48 h. The vomiting was without blood and the abdominal pain started 10 days ago. The pain was in the periumbilical region and was accompanied by fever. She was treated by her family physician with diagnosis of gastroenteritis, with antibiotics (amoxicillin) and antispasmodics (mebeverine). The pain worsened with vomiting and constipation and this brought the patient to the ED. The patient was not using any specific medication and his medical history did not suggest a major disease. Her physical examination revealed a conscious but altered patient: a temperature of 38°C, a pulse rate of 100 per minute and a blood pressure of 100/60 mm Hg. The patient was pale and her abdomen was distended. There was diffuse abdominal and pelvic guarding. The hernial sites were free. On rectal examination, the rectum contained no stool.

Acute abdominal x-ray series showed distended small bowel loops with air fluid levels, and free air under right diaphragm. In view of air under the diaphragm and unstable vital signs, an emergent laparotomy was performed on the patient.

**Figure 1:**
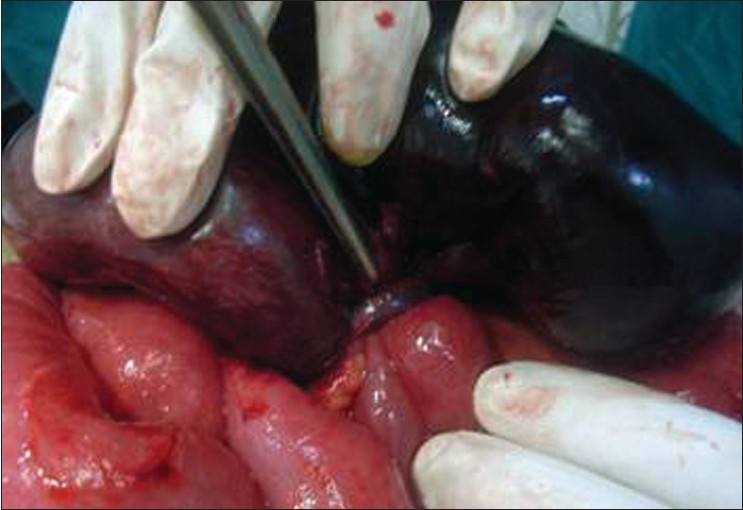
Meckel's diverticulum encircling and strangulating the terminal ileum

**Figure 2:**
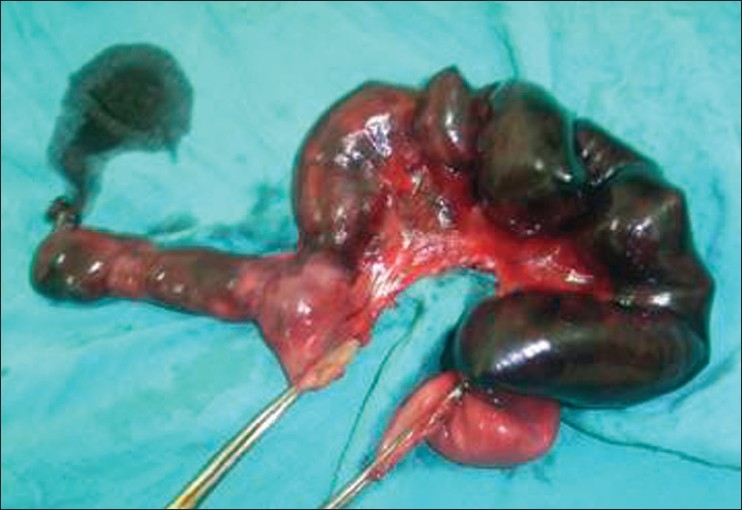
A resection of terminal ileum with perforated giant Meckel's diverticulum

On laparotomy, exploration showed that the peritoneal cavity was filled with 700 cc of free blackish nonclotted blood. There were multiple dilated loops of small bowel and the terminal ileum was gangrenous. At 50 cm from the ileocecal valve, there was a long tubular MD encircling and strangulating the terminal ileum [Figure 1]. It appeared inflamed and perforated, forming an internal hernial orifice as the result of adhesion between an inflammatory end of the diverticulum and the surrounding mesentery. We divided the diverticulum at the mesentery and resected it with 90 cm of ileum [Figure 2].

The pathological examination of the resected specimen showed a Meckel's diverticulum on the antimesenteric border with heterotrophic jejunal mucosa. Postoperatively, the patient failed to improve and died due to sepsis and multi organ failure.

Meckel's diverticulum results from incomplete involution of the most proximal portion of the vitelline or omphalomesenteric duct during the weeks 5-7 of fetal development. The incidence of Meckel's diverticulum in the general population has been estimated to be about 2%.

Only about 4% of patients with MD are symptomatic.[1] The most common symptoms compliment complications such as bleeding, obstruction, inflammation and perforation.

Intestinal obstruction is the most common complication in adult patients.[2] Intussusception is the common presentation of obstruction in patients with MD. Other causes of obstruction include volvulus around fibrous bands adherent to the umbilicus, inflammatory adhesions, Littre's hernias and diverticular strictures.

The second most common complication in adults appears to be related to an inflammatory process. Perforation of the diverticulum usually is secondary to inflammatory diverticulitis and gangrene, although peptic ulceration also can lead to perforation. In our case, the perforation is secondary to inflammatory diverticulitis exacerbated by ischemia and gangrene.

Lower gastrointestinal hemorrhage is the most common presentation in children with a symptomatic Meckel's diverticulum, with incidence rates recorded as high as 50%.[3]

Primary pathological process responsible for bleeding in Meckel's diverticulum is the ileal mucosal ulceration due to the existence of ectopic tissue. Gastric and pancreatic tissues predominate, with corresponding incidences of 60% to 85% and 5% to16%.[4]

From the presentation of our case, we can deduce that our patient first presented with an acute episode of Meckel's diverticulitis. This was followed by adhesion of the diverticulum to the mesentery. Finally, the distal ileum got strangulated in the internal hernia and lead to perforation additionally supported by the existing diverticulitis and ischemia. The hemorrhage resulted from the ulcerations and necrosis.

In conclusion, the association of diverticulitis, small bowel obstruction, perforation and intra-abdominal hemorrhage caused by a large Meckel's diverticulum with the existence of rare heterotrophic jejunal tissue is an exceptional but fatal coincidence in our case. The delayed presentation of the patient was a major factor in her rapid deterioration and death. Early surgical intervention may have possibly saved this young patient.
